# Effect of Oxidative Stress on Homer Scaffolding Proteins

**DOI:** 10.1371/journal.pone.0026128

**Published:** 2011-10-17

**Authors:** Igor Nepliouev, Zhu-Shan Zhang, Jonathan A. Stiber

**Affiliations:** Ion Channel Research Unit and Department of Medicine, Duke University Medical Center, Durham, North Carolina, United States of America; University of Arkansas for Medical Sciences, United States of America

## Abstract

Homer proteins are a family of multifaceted scaffolding proteins that participate in the organization of signaling complexes at the post-synaptic density and in a variety of tissues including striated muscle. Homer isoforms form multimers via their C-terminal coiled coil domains, which allows for the formation of a polymeric network in combination with other scaffolding proteins. We hypothesized that the ability of Homer isoforms to serve as scaffolds would be influenced by oxidative stress. We have found by standard SDS-PAGE of lysates from adult mouse skeletal muscle exposed to air oxidation that Homer migrates as both a dimer and monomer in the absence of reducing agents and solely as a monomer in the presence of a reducing agent, suggesting that Homer dimers exposed to oxidation could be modified by the presence of an inter-molecular disulfide bond. Analysis of the peptide sequence of Homer 1b revealed the presence of only two cysteine residues located adjacent to the C-terminal coiled-coil domain. HEK 293 cells were transfected with wild-type and cysteine mutant forms of Homer 1b and exposed to oxidative stress by addition of menadione, which resulted in the formation of disulfide bonds except in the double mutant (C246G, C365G). Exposure of myofibers from adult mice to oxidative stress resulted in decreased solubility of endogenous Homer isoforms. This change in solubility was dependent on disulfide bond formation. *In vitro* binding assays revealed that cross-linking of Homer dimers enhanced the ability of Homer 1b to bind Drebrin, a known interacting partner. Our results show that oxidative stress results in disulfide cross-linking of Homer isoforms and loss of solubility of Homer scaffolds. This suggests that disulfide cross-linking of a Homer polymeric network may contribute to the pathophysiology seen in neurodegenerative diseases and myopathies characterized by oxidative stress.

## Introduction

Homer proteins are a family of multifaceted scaffolding proteins that share a highly conserved Ena/VASP Homology 1 (EVH1) domain at their amino termini which allows binding to proline-rich motifs on Homer ligands which include group I metabotropic glutamate receptors, inositol triphosphate receptors (IP3R), the actin-binding protein Drebrin, and several members of the transient receptor potential (TRP) channel family [1], [2], [3]. Constitutively expressed Homer isoforms such as Homer 1b and 1c, in addition to containing an amino-terminal EVH1 domain, also contain a C-terminal coiled-coil domain allowing Homer proteins to self-multimerize [4]. Homer 1a, which was identified as an immediate early gene (IEG), lacks a C-terminal coiled-coil domain [5]. The different isoforms of the three identified Homer genes (Homer 1, 2, and 3) are the result of alternative splicing [6]. Based on recently published crystallographic analysis of Homer 1 isoforms, Homer proteins form dimers via leucine zipper motifs at their C-terminal coiled-coil domains [7]. Two dimers can then intercalate in a tail-to-tail fashion to form a tetramer. Homer tetramers form a polymeric network structure at the post synaptic density (PSD) through their interaction with other scaffolding proteins such as Shank, and this network is required for maintenance of dendritic spine structure and synaptic function [7]. A scaffolding protein complex involving Homer and Shank provides spatial organization to proteins involved in calcium signaling and links proteins involved in endocytosis and receptor recycling such as dynamin-3 to the PSD [1], [8].

Homer 1 interacts with several members of the transient receptor potential (TRP) channel family which have been implicated in the abnormal calcium influx noted in muscle fibers from dystrophic mice [2]. We previously reported that mice lacking Homer 1 exhibited a myopathy characterized by smaller muscle fiber cross-sectional area and decreased skeletal muscle force generation which was associated with dysregulation of TRP channel activity [9]. Regulation of Homer scaffolds has previously been shown to occur via both transcriptional control and post-translational modification. Homer 1a was first identified as an immediate early gene (IEG) whose expression was rapidly upregulated in the rat brain after seizure activity [5]. Homer 3 isoforms, which predominate in Purkinje neurons, have been shown to be phosphorylated by calcium/calmodulin-dependent kinase II (CamKII) resulting in dissociation of these isoforms from the metabotropic glutamate receptor 1α [10]. Regulation of Homer scaffolds by redox mechanisms has not previously been described. We have found by standard SDS-PAGE of adult mouse skeletal muscle lysates exposed to air oxidation that Homer migrates as both a dimer and monomer in the absence of reducing agents and solely as a monomer in the presence of a reducing agent such as tris (2-carboxyethyl) phosphine (TCEP) or beta-mercaptoethanol (BME). This serendipitous observation led us to the hypothesis that Homer scaffolds could be regulated by oxidation.

## Results

**Figure 1:**
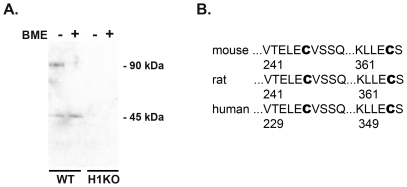
Immunoblotting of skeletal muscle lysates exposed to air oxidation. A) Western blot of adult mouse skeletal muscle lysates showing that Homer migrates as both a dimer and monomer in the absence of reducing agent (lane 1) and solely as a monomer in the presence of reducing agent (BME, lane 2). Neither the 90 kDa or 45 kDa band was observed in skeletal muscle lysates from Homer 1 knockout mice. B) Homer 1b protein sequence showing the location of the two cysteine residues (residues 246 and 365).

We found by standard SDS-PAGE of adult mouse skeletal muscle lysates exposed to air oxidation that Homer migrates as both a dimer and monomer in the absence of reducing agents and solely as a monomer in the presence of a reducing agent such as *tris* (2-carboxyethyl) phosphine (TCEP) or beta-mercaptoethanol (BME) (Figure 1A). In the absence of a reducing agent, Western blotting of adult skeletal muscle lysates exposed to air oxidation using a pan-Homer antibody revealed the presence of bands at 90 kDa and 45 kDa corresponding to a Homer dimer and monomer respectively. In the presence of reducing agent, a single band of 45 kDa corresponding to a Homer monomer was observed. Similar results were observed with protein lysates generated from C2C12 myotubes or brain tissue (not shown). Neither the 90 kDa or 45 kDa band was observed in a skeletal muscle lysates from Homer 1 knockout mice which confirmed the specificity of these bands and was consistent with our previous observation that Homer 1 isoforms are the predominant Homer isoforms in muscle (Figure 1A) [9]. Homer proteins have previously been shown to form dimers via leucine zipper motifs contained within their C-terminal coiled-coil domains [7], [11]. Our finding suggested that Homer dimers exposed to oxidation could be stabilized by the presence of an inter-molecular disulfide bond. Analysis of the entire peptide sequence of Homer 1b revealed the presence of only two cysteine residues, both of which were located in conserved regions adjacent to the C-terminal coiled-coil domains: one cysteine at position 246 and a second cysteine at position 365 directly adjacent to the C-terminal amino acid (Figure 1B).

**Figure 2:**
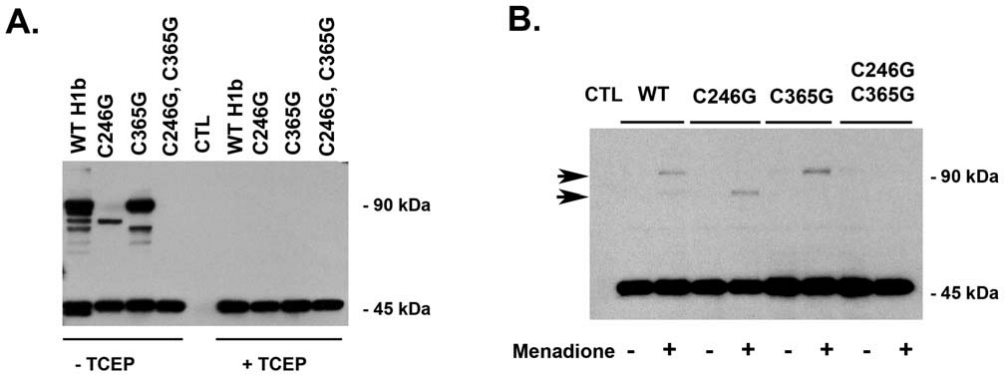
Oxidation results in disulfide cross-linking of Homer dimers. A) WT and mutant forms of Homer 1b were expressed in HEK 293 cells and exposed to air oxidation post lysis. WT and single cysteine mutants showed evidence of disulfide bond formation and migrated as dimers with varying mobility. With mutation of both cysteine residues (C246G, C365G) or addition of the reducing agent TCEP, Homer 1b migrated solely as a monomer. No bands were observed in lysates from untransfected control cells (CTL). B) HEK 293 cells were transfected with WT and mutant forms of Homer 1b, and cells were exposed to oxidative stress by addition of 200 µM menadione for 10 min. Oxidative stress resulted in the formation of disulfide bonds except in the double mutant (C246G, C365G). No bands were observed in lysates from untransfected control cells (CTL).

Wild-type (WT) and mutant forms of Homer 1b were tagged with the V5 epitope, expressed in HEK 293 cells, and exposed to air oxidation after cell lysis. WT and single cysteine mutants (C246G or C365G) exposed to air oxidation migrated as dimers with varying mobility based on the presence or absence of specific cysteine residues (Figure 2A). The ability of WT and single cysteine mutants exposed to air oxidation to migrate as bands of multiple sizes under denaturing conditions was consistent with the presence of multiple disulfide-linked structures. The dimer bands of greatest intensity were consistently observed for WT Homer 1b and the C365G mutant: suggesting that the cysteine residue at position 246 showed greater availability for the formation of disulfide bonds. With mutation of both cysteine residues (C246G, C365G) or addition of the reducing agent TCEP, Homer 1b migrated solely as a monomer. For WT Homer 1b a doublet monomer band was also observed which was not seen with mutation of either or both cysteine residues. The likely explanation of this doublet monomer band is that in the presence of two cysteine residues, the formation of an intramolecular disulfide bond between cysteine residues of a single Homer monomer is possible which would affect mobility by electrophoresis. No bands were observed in lysates from untransfected control cells (CTL) (Figure 2A). To assess the effects of intracellular oxidative stress, HEK 293 cells were transfected with WT and mutant forms of Homer 1b and exposed to oxidative stress by the addition of menadione (200 µM), a model redox cycling quinone, for 10 minutes [12]. Following stimulation, cells were lysed in the presence of n-ethylmaleimide which alkylates free cysteine residues in order to block air oxidation post-lysis. Menadione stimulation resulted in dimer formation with varying mobility based on the presence or absence of specific cysteine residues, consistent with the formation of disulfide bonds except in the double mutant (C246G, C365G)(Figure 2B).

**Figure 3:**
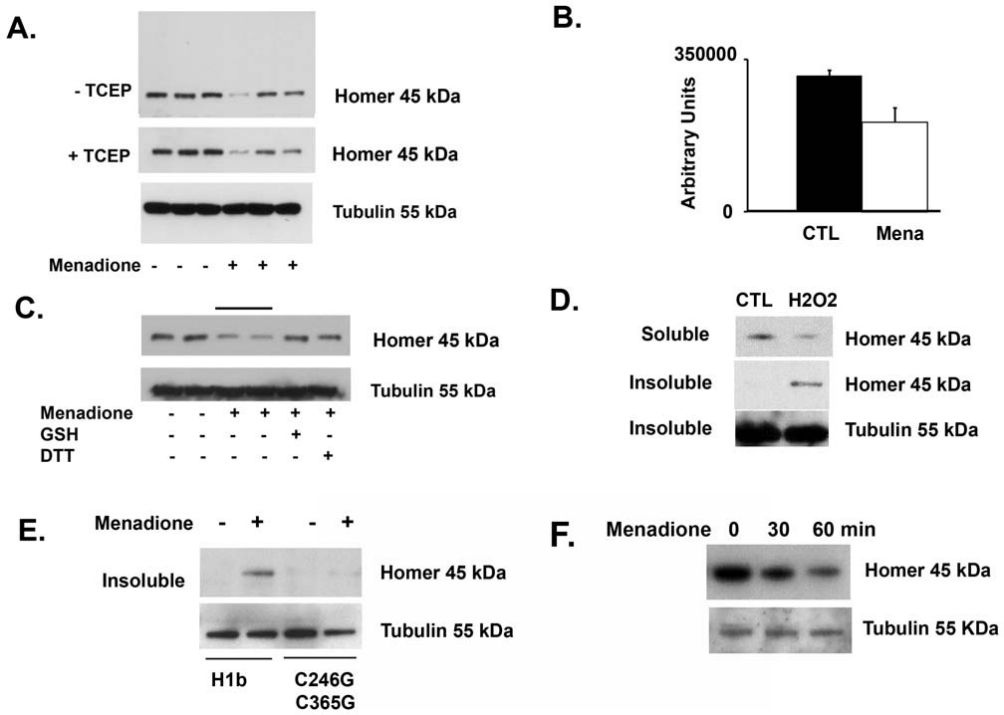
Oxidative modification of Homer results in loss of solubility. A) Western blot of lysates from C2C12 myotubes under control conditions (-) and conditions of oxidative stress (+200 µM menadione for 30 min.). Myotubes were lysed in 1% Triton and in the presence of 20 mM n-ethylmaleimide and run under non-reducing (−TCEP) and reducing conditions (+TCEP). B) Quantification of detectable Homer expression under control conditions (CTL) and after exposure to menadione (Mena) by non-reducing Western blot shown in (A). C) The addition of a reducing agent, either 10 mM reduced glutathione or 10 mM DTT, to the media prior to menadione exposure successfully blocked the loss in detectable Homer observed after prolonged (1 hour) exposure to menadione (Figure 3C). D) Adult myofibers were exposed to control conditions or oxidative stress by addition of 200 µM H_2_O_2_. Western blot of Homer protein expression showing a decrease in detectable Homer in the Triton soluble fraction in response to oxidative stress and an increase in Homer detected in Triton insoluble fraction. E) Lysates from HEK 293 cells transfected with WT Homer 1b or the double mutant (C246G, C365G) and exposed to intracellular oxidative stress (200 µM menadione for 30 min.) were separated into Triton soluble and insoluble fractions. WT Homer 1b was detected in the Triton insoluble fraction only in response to oxidative stress, but no evidence of the double mutant (C246G, C365G) was detected in the insoluble fraction in response to oxidative stress. F) C2C12 myotubes were exposed to 200 µM menadione for 0, 30, and 60 minutes respectively. Cells were lysed in buffer containing 1% Triton, 8 M guanidine HCL, and 50 mM TCEP. Analysis of whole lysates showed a decrease in detectable Homer over time.

We then investigated the effect of oxidative stress on endogenous Homer isoforms in C2C12 myotubes. Unexpectedly, we did not observe any change in the migration of endogenous Homer isoforms with oxidative stress under non-reducing conditions. However, exposure of C2C12 myotubes to oxidative stress in the form of 200 µM menadione did result in a significant decrease in the amount of Homer detectable by immunoblotting in the Triton soluble fractions (Figure 3A–B). This decrease in detectable Homer expression was time dependent with less Homer detected after longer exposures to menadione (not shown). After exposure of C2C12 myotubes, adult myofibers, or adult cardiomyocytes to oxidative stress (200 µM menadione or H_2_O_2_), we consistently found that the decrease in detectable Homer in the Triton soluble fraction in response to oxidative stress was associated with an increase in Homer detected in Triton insoluble fraction: under basal conditions no Homer was detected in the insoluble fraction (Figure 3D). The addition of a reducing agent such as 10 mM DTT or reduced glutathione, to the media prior to menadione exposure successfully blocked the loss in Homer solubility observed after prolonged (1 hour) exposure to menadione (Figure 3C). To determine if the change in Homer solubility in response to oxidative stress was dependent on cysteine residues, lysates from HEK 293 cells transfected with WT Homer 1b or the double mutant (C246G, C365G) and exposed to intracellular oxidative stress were separated into Triton soluble and insoluble fractions. This change in solubility was dependent of the presence of cysteine residues as we observed the presence of WT Homer 1b in the Triton insoluble fraction only in response to oxidative stress, and we did not observe any evidence of the double mutant (C246G, C365G) in the insoluble fraction in response to oxidative stress (Figure 3E). Single cysteine mutants, as well, were observed in the insoluble fraction only in response to oxidative stress (not shown). We also examined the time-dependence of detectable Homer expression in whole lysates from myotubes exposed to varying durations of oxidative stress. C2C12 myotubes were exposed to 200 µM menadione for 0, 30, and 60 minutes respectively. Myotubes were lysed in buffer containing 1% Triton, 8 M guanidine hydrochloride, and 50 mM TCEP or buffer containing 4% SDS, 8 M urea, and 50 mM TCEP to maximize protein solubility. Analysis of whole lysates showed a decrease in detectable Homer over time after exposure to oxidative stress under both of these lysis conditions (Figure 3F).

**Figure 4:**
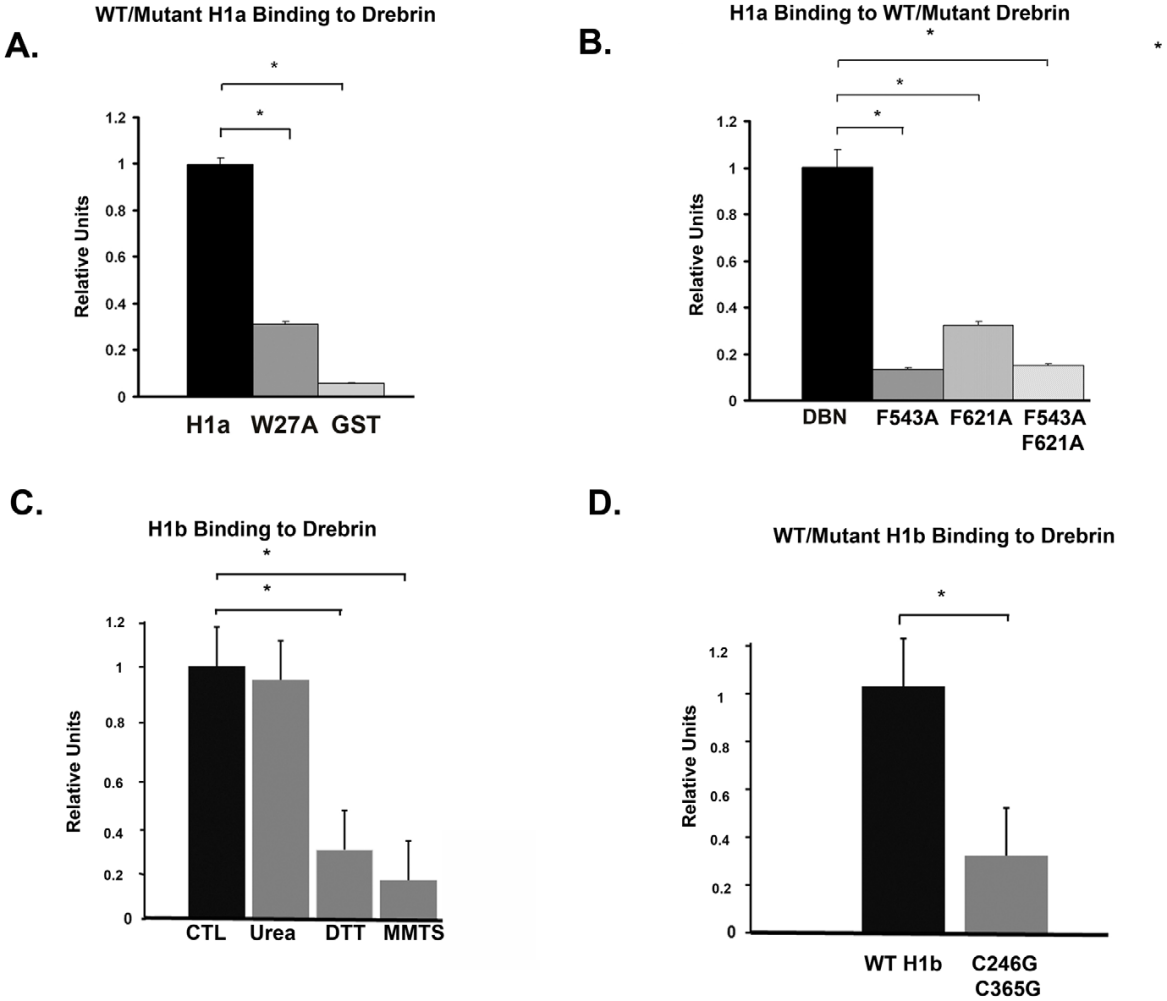
Effect of oxidative modification on the *in vitro* interaction of Homer and Drebrin. A) Recombinant Homer 1a was expressed as a GST fusion protein in E. coli and purified. We saw a significant *in vitro* interaction between recombinant Homer 1a and recombinant Drebrin using an ELISA format. GST protein alone served as a negative control and showed an insignificant interaction with Drebrin. W27A mutation of the EVH1 domain of Homer 1a significantly inhibited the interaction of Homer 1a-GST and Drebrin. B) Mutation of the first (F543A) or second (F621A) or both (F543A, F621A) Homer binding motifs (PPxxF) at the carboxyl terminus of Drebrin significantly inhibited the interaction of recombinant Homer 1a and Drebrin *in vitro* as measured by ELISA. C) *In vitro* binding of recombinant Homer 1b to Drebrin. Urea (2 M) had no effect on binding, while reduction of disulfide bonds with DTT (50 mM) or S-methylthiolation of cysteine resides with MMTS (10 mM) significantly blocked binding of recombinant Homer 1b to Drebrin. D) Mutation of both cysteine residues of Homer 1b resulted in a significant decrease in binding to Drebrin compared to WT Homer 1b.

We sought to determine the effect of disulfide cross-linking on the ability of Homer dimers to interact with their binding partners. Drebrin represents a model Homer interacting partner because of the presence of two Homer binding motifs at its C-terminus and has previously been used by *in vitro* assays to determine the effect of phosphorylation of Homer isoforms on their ability to bind ligands [10]. We confirmed the interaction between Homer 1 isoforms and Drebrin using an *in vitro* binding assay in an ELISA format and validated the specificity of this interaction using mutational analysis. Recombinant Homer 1a was expressed as a GST fusion protein in BL21 E. coli and purified. We saw a significant *in vitro* interaction between recombinant Homer 1a and recombinant human Drebrin by ELISA. GST protein alone served as a negative control and showed an insignificant interaction with Drebrin. W27A mutation of the EVH1 domain of Homer 1a, which has previously been shown to disrupt binding through this domain, significantly inhibited the interaction of Homer 1a-GST and Drebrin (Figure 4A) [2]. Drebrin contains two Homer binding motifs (PPxxF) which are highly conserved [3], [10]. Mutation of the first (F543A) or second (F621A) or both (F543A, F621A) Homer binding motifs at the carboxyl terminus of Drebrin significantly inhibited the *in vitro* interaction of these recombinant proteins (Figure 4B). Thus, our mutational analysis confirmed that binding between recombinant Homer 1 isoforms and Drebrin depends on an interaction between the amino terminal EVH1 domain and Homer binding motifs (PPxxF) at the carboxyl terminal end of Drebrin (Figure 4B).

The interaction between Homer isoforms and Drebrin occurs does not require the presence of the C-terminal coiled-coil domain, as Homer 1a lacks this domain. However, cysteine residues adjacent to the C-terminal coiled-coil domain of Homer 1b influenced the interaction with Drebrin. Homer 1b was also able to interact with Drebrin by *in vitro* binding assays, but we found that the oxidation state of the cysteine residues of the C-terminus of Homer 1b influenced the stability of the Homer-Drebrin interaction. Recombinant Homer 1b, which had been exposed to air oxidation, was treated with either urea, the reducing agent dithiothreitol (DTT), or dithiothreitol followed by methyl methanethiosulfonate (MMTS), which modifies sulfhydryl groups on cysteine residues, to determine the effects on Homer-Drebrin binding. These substances were removed with a desalting column (Millipore, 10K Amicon Ultra-4) prior to addition of recombinant Homer 1b to the microtiter plate. Urea (2 M) had no effect on binding, but reduction of disulfide bonds with DTT (50 mM) or S-methylthiolation of cysteine resides with methylmethane thiosulfate (MMTS 10 mM) significantly blocked binding of recombinant Homer 1b to Drebrin (Figure 4C). Mutation of both cysteine residues of Homer 1b (C246G, C365G) resulted in a significant decrease in binding to Drebrin compared to WT Homer 1b (Figure 4D). These *in vitro* binding data are consistent with disulfide cross-linking of Homer dimers enhancing the interaction between Homer 1b and Drebrin.

## Discussion

Here, we provide the first description of oxidative modification of Homer scaffolds. As the Homer scaffolding network has been shown to influence the spatial organization and regulation of signaling proteins in myocytes and at the PSD of neurons, this has important implications for the pathophysiology of disorders characterized by oxidative stress [7], [9]. SDS-PAGE of lysates from adult mouse skeletal muscle exposed to air oxidation revealed that Homer migrates as both a dimer and monomer in the absence of reducing agents and solely as a monomer in the presence of a reducing agent: suggesting that Homer dimers exposed to oxidation could be modified by the presence of an inter-molecular disulfide bond (Figure 1A). WT and single cysteine mutants (C246G or C365G) of Homer 1b which were heterologously expressed in HEK 293 cells and exposed to air oxidation migrated as dimers with varying mobility based on the presence or absence of specific cysteine residues (Figure 2A). The presence of multiple bands of varying mobility when WT Homer 1b is overexpressed and exposed to air oxidation is likely due to formation of multiple combinations of disulfide bonds between Homer monomers that is only possible when two cysteine residues are present (i.e. C246-C246 in a parallel or anti-parallel conformation, C246–C365 in a parallel or anti-parallel conformation, C365-C365 in a parallel or anti-parallel conformation). Hayashi et. al. recently reported that Homer proteins form parallel dimers via leucine zipper motifs at their C-terminal coiled-coil domains [7]. Two dimers can then intercalate in a tail-to-tail (anti-parallel) fashion to form a tetramer. Our results are consistent with this model. With mutation of only a single cysteine residue, disulfide bond cross-linking of a Homer dimer is still possible through the remaining intact cysteine residue. The intensity of bands corresponding to cross-linked Homer dimers was higher with air oxidation *ex vivo* than with exposure to living cells to menadione. This was not unexpected given the highly reductive intracellular environment of cultured cells [13].

Exposure of heterologously expressed Homer 1b to oxidative stress resulted in disulfide cross-linking of Homer dimers, while exposure of endogenous Homer to oxidative stress resulted in decreased solubility of Homer scaffolds in C2C12 myotubes, adult skeletal myofibers, and adult cardiomyocytes. The contrast between the effects of oxidative stress on heterologously expressed Homer isoforms and endogenous Homer isoforms likely reflects differences in spatial organization between heterologous and endogenous Homer expression. Heterologously expressed Homer isoforms can form dimers through their coiled-coil domains which can then be cross-linked by exposure to oxidative stress. Endogenous Homer isoforms are more likely to exist as part of a network comprised of higher order structures: tetramers which form a network through interactions with other scaffolding proteins [7]. Exposure of endogenous Homer scaffolds to oxidative stress would therefore be likely to result in extensive cross-linking of these higher order structures and decreased solubility. A decrease in endogenous Homer in C2C12 myotubes exposed to oxidative stress was detected by Western blotting under both reducing and non-reducing conditions (Figure 3A–B). Addition of a reducing agent did block the reduction in detectable Homer in the soluble fraction when added to the media prior to exposure of myotubes to oxidative stress however (Figure 3C). This was consistent with our subsequent finding that the decrease in detectable Homer was associated with a loss of solubility that was dependent on disulfide cross-linking: pre-treatment with a reducing agent would be expected to block disulfide cross-linking and thus prevent the change in solubility that resulted in loss of detectable Homer in the soluble fraction.

Oxidative modification of Homer scaffolds may result in the formation of a variety of possible disulfide bonds: between individual components of a Homer dimer, between two Homer dimers to form higher order structures, or mixed disulfide bonds with other proteins or low-molecular-weight thiol compounds such as cysteine and oxidized glutathione. An increase in detectable Homer was observed in the Triton insoluble fraction after exposure to oxidative stress. This decrease in solubility can result from misfolding of cross-linked higher order structures, and these insoluble cross-linked Homer scaffolds would be susceptible to degradation by the proteosome [14]. We observed a significant time-dependent decrease in detectable Homer in whole lysates after exposure to oxidative stress consistent with degradation of misfolded proteins (Figure 3F).

The Homer – Drebrin interaction is mediated by the EVH1 domain at the N-terminus of Homer isoforms and two Homer binding motifs at the C-terminus of Drebrin [3]. Nevertheless, post-translational modification of residues outside of the Homer EVH1 domain has previously been shown to regulate the interaction of Homer isoforms and Drebrin. Mizutani et. al. previously demonstrated that phosphorylation of Homer 3 by CamKII resulted in decreased affinity of Homer 3 isoforms for the C-terminus of Drebrin [10]. All the serine residues phoshorylated by CamKII were located downstream of the N-terminal EVH1 domain. While the C-terminal coiled-coil domain of Homer 1 isoforms is not required for the Homer 1-Drebrin interaction, we provide evidence that the disulfide cross linking of Homer dimers via their C-terminus stabilizes their interaction with Drebrin. Drebrin contains two Homer binding motifs (PPxxF) at the C-terminus which are highly conserved [3], [10]. It is likely that Homer dimers bind more efficiently to Drebrin than monomers, and disulfide cross-linking locks Homer dimers in a conformation which is favorable for binding to Drebrin. Although resulting in enhanced binding to Drebrin, cross-linking of Homer dimers would be expected to result in a loss of plasticity.

Oxidative stress resulted in decreased solubility of endogenous Homer isoforms through cross-linking of critical cysteine residues. This decrease in solubility would be expected to result in loss of Homer scaffolds through degradation of misfolded proteins [14]. As Homer scaffolds are enriched at the PSD, this provides a potential mechanism for why the PSD is highly susceptible to oxidative stress such as is observed in several neurodegenerative disorders such as Alzheimer's disease, Parkinson's disease, and traumatic brain injury [15], [16], [17], [18]. As Homer scaffolds form polymeric network structures, oxidative damage to a Homer network has implications for other scaffolding proteins at the PSD such as Shank which participate in the formation of this network [7]. Proteins which participate in the formation of a network structure with Homer may be susceptible to changes in solubility through their association with this network. Oxidative modification of Homer scaffolds also has implications for signaling proteins which interact with Homer such as group I metabotropic glutamate receptors, inositol triphosphate receptors (IP3R) and several members of the transient receptor potential (TRP) channel family [1], [2]. Since loss of Homer 1 isoforms has been shown to result in TRP channel dysregulation in skeletal muscle, oxidative modification of Homer scaffolds may also provide a link between oxidative stress and dysregulation of TRP channel activity such as that observed in Duchenne's muscular dystrophy [19], [20], [21], [22].

## Materials and Methods

### Ethics Statement

Experiments involving mice were performed according to NIH policies outlined in the Guide for Care and Use of Laboratory Animals. All protocols for animal research were reviewed by the Institutional Animal Care and Use Committee (IACUC) of Duke University Medical Center (Duke University IACUC protocol # A124-10-05).

### Cloning and Mutational Analysis of Homer 1 isoforms

PCR primers were used to amplify the open reading frames of Homer 1b from adult mouse skeletal muscle as described by Sandona et. al. [23]. Homer 1b was then subcloned into the pEF-V5 vector (Invitrogen) using the EcoRI restriction site, and the correct orientation was confirmed by sequencing. Site-directed cysteine mutants of Homer 1b were generated using a PCR based approach and verified by direct sequencing.

### Western blotting and Triton Fractionation

Prior to protein analysis by Western blotting, cells were lysed in 1% Triton lysis buffer containing 1% Triton X-100, 150 mM NaCl, 50 mM Tris, pH 8, and protease inhibitor (Sigma) unless otherwise specified. N-ethylmaleimide (20 mM) was added to the lysis buffer where indicated to block protein oxidation post-lysis. Protein concentrations were measured by the Bradford assay or by Coomassie staining after SDS-polyacrylamide gel electrophoresis (PAGE) to ensure equal loading. Protein transfer was confirmed by Ponceau staining, and equal loading was confirmed by immunoblotting for α-tubulin (Developmental Studies Hybridoma Bank, University of Iowa). Detection of endogenous Homer isoforms by Western blotting was performed using a pan-Homer antibody (Santa Cruz Biotechnology). Analysis of heterologous expression of V5-tagged Homer isoforms and cysteine mutants was performed using a monoclonal antibody which recognizes the V5 tag (Invitrogen). For separation of Triton soluble and insoluble fractions, whole lysates were centrifuged at 10,000 g for 30 minutes at 4°C. The supernatant served as the soluble fraction while the pellet (insoluble fraction) was then washed three times with PBS and resuspended in lysis buffer containing 4% SDS, 8 M urea, and protease inhibitor.

### Cell Culture, Transfection, Myofiber Isolation

HEK 293 cells (ATCC, Manassas, VA) were transfected with wild type and mutant Homer isoforms using Fugene reagent (Promega). C2C12 cells (ATCC) were grown in Dulbecco's modified media (DMEM) (Gibco) supplemented with 20% fetal bovine serum and 100 IU/ml penicillin and 100 µg/ml streptomycin. Cell differentiation into myotubes took place over 4 days while cultured in differentiation media (DM) containing DMEM containing 2% horse serum, 100 IU/ml penicillin and 100 µg/ml streptomycin, 10 µg/ml transferrin, 10 µg/ml regular insulin, and 50 mM HEPES buffer. Experiments involving C2C12 cells were performed after 4 days in DM except when otherwise noted. Adult skeletal myofibers were isolated from the flexor digitorum brevis muscle of Homer 1 KO mice and wild type littermate controls by collagenase digestion (Type I, Worthington) and tituration using a glass pipet. Adult cardiomyocytes from WT and Homer 1 KO mice were prepared using Langendorf perfusion of hearts with low Ca^2+^ collagenase containing solution to isolate single cells [24].

### *In Vitro* Binding Assays

Recombinant proteins representing the wild-type and mutant forms of Homer 1 isoforms and Drebrin (human full length cDNA, Origene) were expressed in BL21 E. coli and purified via their 6-His tags using on a Ni^2+^-agarose column. As indicated, wild type or mutant forms of recombinant Drebrin were adsorbed to wells of a microtiter plate. Non-specific binding was blocked with 5% milk in phosphate buffered saline (PBS) containing 0.1% Tween for 5 hours at room temperature. Recombinant Homer isoforms or a glutathione-S-transferase (GST) control were then added to the microtiter wells in blocking solution and incubated 2 hours. After incubation, the wells were washed four times with 0.05% Tween in PBS. Homer-Drebrin binding was determined by an ELISA with a rat anti-Homer antibody (1∶8000 dilution, Chemicon) followed by a mouse ant-rat HRP-conjugated antibody (1∶5000 dilution, Jackson ImmunoResearch). After incubation with secondary antibody, wells were again washed four times with 0.05% Tween in PBS. The signal was then developed with chemiluminescence substrate (ECL Advance, GE Amersham) and measured on a lumenometer. After subtraction of background signal, the concentration of wild-type and mutant Drebrin adsorbed on the microtiter plate was determined with a V5 monoclonal antibody and was used for normalization of results. Each measurement of *in vitro* binding was performed in triplicate. For experiments in which the effect of urea, DTT, or MMTS on Homer-Drebrin binding were assessed, these substances were removed with a desalting column (Millipore, 10K Amicon Ultra-4) prior to addition to the microtiter plate.

### Statistical Analysis

Data are presented as means ± standard errors of the means. An unpaired Student's *t* test was used for comparison between two groups. Values of *P* of <0.05 were considered significant.

## References

[1] Tu JC, Xiao B, Yuan JP, Lanahan AA, Leoffert K (1998). Homer binds a novel proline-rich motif and links group 1 metabotropic glutamate receptors with IP3 receptors.. Neuron.

[2] Yuan JP, Kiselyov K, Shin DM, Chen J, Shcheynikov N (2003). Homer binds TRPC family channels and is required for gating of TRPC1 by IP3 receptors.. Cell.

[3] Shiraishi-Yamaguchi Y, Sato Y, Sakai R, Mizutani A, Knopfel T (2009). Interaction of Cupidin/Homer2 with two actin cytoskeletal regulators, Cdc42 small GTPase and Drebrin, in dendritic spines.. BMC Neuroscience.

[4] Xiao B, Tu JC, Petralia RS, Yuan JP, Doan A (1998). Homer regulates the association of group 1 metabotropic glutamate receptors with multivalent complexes of homer-related, synaptic proteins.. Neuron.

[5] Brakeman PR, Lanahan AA, O'Brien R, Roche K, Barnes CA (1997). Homer: a protein that selectively binds metabotropic glutamate receptors.. Nature.

[6] Bottai D, Guzowski JF, Schwarz MK, Kang SH, Xiao B (2002). Synaptic activity-induced conversion of intronic to exonic sequence in Homer 1 immediate early gene expression.. Journal of Neuroscience.

[7] Hayashi MK, Tang C, Verpelli C, Narayanan R, Stearns MH (2009). The postsynaptic density proteins Homer and Shank form a polymeric network structure.. Cell.

[8] Lu J, Helton TD, Blanpied TA, Racz B, Newpher TM (2007). Postsynaptic positioning of endocytic zones and AMPA receptor cycling by physical coupling of dynamin-3 to Homer.. Neuron.

[9] Stiber JA, Zhang ZS, Burch J, Eu JP, Zhang S (2008). Mice lacking Homer 1 exhibit a skeletal myopathy characterized by abnormal transient receptor potential channel activity.. Molecular & Cellular Biology.

[10] Mizutani A, Kuroda Y, Futatsugi A, Furuichi T, Mikoshiba K (2008). Phosphorylation of Homer3 by calcium/calmodulin-dependent kinase II regulates a coupling state of its target molecules in Purkinje cells.. Journal of Neuroscience.

[11] Irie K, Nakatsu T, Mitsuoka K, Miyazawa A, Sobue K (2002). Crystal structure of the Homer 1 family conserved region reveals the interaction between the EVH1 domain and own proline-rich motif.. J Mol Biol.

[12] Thor H, Smith MT, Hartzell P, Bellomo G, Jewell SA (1982). The metabolism of menadione (2-methyl-1,4-naphthoquinone) by isolated hepatocytes. A study of the implications of oxidative stress in intact cells.. Journal of Biological Chemistry.

[13] Malinouski M, Zhou Y, Belousov VV, Hatfield DL, Gladyshev VN (2011). Hydrogen peroxide probes directed to different cellular compartments.. PLoS ONE [Electronic Resource].

[14] Brodsky JL (2010). The use of in vitro assays to measure endoplasmic reticulum-associated degradation.. Methods Enzymol.

[15] Ansari MA, Scheff SW (2010). Oxidative stress in the progression of Alzheimer disease in the frontal cortex.. J Neuropathol Exp Neurol.

[16] Butterfield DA, Perluigi M, Sultana R (2006). Oxidative stress in Alzheimer's disease brain: new insights from redox proteomics.. Eur J Pharmacol.

[17] Rabinovic AD, Lewis DA, Hastings TG (2000). Role of oxidative changes in the degeneration of dopamine terminals after injection of neurotoxic levels of dopamine.. Neuroscience.

[18] Ansari MA, Roberts KN, Scheff SW (2008). Oxidative stress and modification of synaptic proteins in hippocampus after traumatic brain injury.. Free Radical Biology & Medicine.

[19] Gervasio OL, Whitehead NP, Yeung EW, Phillips WD, Allen DG (2008). TRPC1 binds to caveolin-3 and is regulated by Src kinase - role in Duchenne muscular dystrophy.. J Cell Sci.

[20] Tidball JG, Wehling-Henricks M (2007). The role of free radicals in the pathophysiology of muscular dystrophy.. Journal of Applied Physiology.

[21] Williams IA, Allen DG (2007). The role of reactive oxygen species in the hearts of dystrophin-deficient mdx mice.. American Journal of Physiology - Heart & Circulatory Physiology.

[22] Ducret T, Vandebrouck C, Cao ML, Lebacq J, Gailly P (2006). Functional role of store-operated and stretch-activated channels in murine adult skeletal muscle fibres.. J Physiol.

[23] Sandona D, Tibaldo E, Volpe P (2000). Evidence for the presence of two homer 1 transcripts in skeletal and cardiac muscles.. Biochem Biophys Res Commun.

[24] Seth M, Zhang ZS, Mao L, Graham V, Burch J (2009). TRPC1 Channels Are Critical for Hypertrophic Signaling in the Heart.. Circ Res.

